# Melkersson-Rosenthal Syndrome with Orofacial Swelling and Recurrent Lower Motor Neuron Facial Nerve Palsy: A Case Report and Review of the Literature

**DOI:** 10.1155/2015/214946

**Published:** 2015-11-09

**Authors:** Jerome Okudo, Yemi Oluyide

**Affiliations:** ^1^School of Public Health, University of Texas, 1200 Pressler Street, Houston, TX 77030, USA; ^2^Warrawong Accident and Medical Center, King Street, Westfield, Warrawong, NSW 2502, Australia

## Abstract

Melkersson-Rosenthal Syndrome (MRS) is a rare otoneurologic condition, which is poorly understood and often underdiagnosed. Etiology and incidence are unclear, although infectious, inflammatory, and genetic causes have been implicated. Recurrent facial nerve palsy, facial swelling, and fissured tongue are the symptoms and signs of this condition. However, this triad is not typical in all patients as patients may present with one or more of the symptoms, which makes management of this condition difficult. Steroids may prove to be useful especially in patients who have facial nerve palsy. In this case report, we have described a 46 year-old Caucasian male who presented to the clinic for the evaluation of orofacial swelling and left facial deviation with a history of multiple treatments for recurrent lower motor neuron type facial nerve palsy.

## 1. Introduction

Melkersson-Rosenthal Syndrome (MRS) in its classic form presents with a triad of recurrent facial nerve palsy, facial edema, and fissured tongue [1–5]. The term was coined between 1928 and 1931 when it was described initially with orofacial edema and facial palsy in 1928 and fissured tongue in 1931, respectively. It is a rare disorder and may receive poor attention and misdiagnosis [1, 6, 7]. This clinical triad is found in less than a third of patients [3, 8]. Some authors have described this condition as a diagnosis of exclusion [2, 9]. Cases have been reported in the continents of Africa, Asia, Australia, Europe, and America and there is no racial or gender predilection [3, 5, 6, 8, 10]. This condition does not have a specific duration or timeline and its prognosis has not been well studied. It has not been proven to have a genetic, infectious, or inflammatory basis; however migraine headaches and autoimmune disorders have been linked to this condition [1, 3, 6, 11]. Patients may present to the otolaryngology, neurology, or dermatology clinic [4, 5, 8].

## 2. Case Presentation

A 46-year-old Caucasian male presented to the outpatient clinic for evaluation of right-sided facial numbness over the ophthalmic (V1) and maxillary division (V2), inability to close the right eye, inability to wrinkle the right side of the forehead, left facial deviation suggestive of a lower motor neuron type facial palsy, and painless swelling of the upper lip and right side of the face for five days. This patient had been treated for recurrent lower motor neuron type facial palsy in the preceding two years prior to presentation with oral steroids, and the patient's symptoms improved each time he was treated. There was no family history of similar complaints and there was no fissured tongue. There was no personal or family history of sarcoidosis or Crohn's disease. On examination, he was found to have upper lip swelling, right facial fullness, right facial paresthesia, and an isolated right-sided facial nerve paralysis with a blood pressure of 220/120 mmHg. He was referred to the emergency department for further investigation and management; however, his symptoms remained. Chest X-ray, renal duplex ultrasound, colonoscopy, and brain CT scan were performed to rule out sarcoidosis, renal artery disease, Crohn's disease, and stroke. We could not perform an MRI brain with contrast with constructive interference in steady state (CISS) with a focus on the 7th nerve course because of the patient's medical insurance type; however, we ruled out pachymeningitis as the patient did not present with complaints of chronic headaches, facial pain, loss of vision, or severe vomiting.

He was empirically managed with 50 mg prednisone daily for five days, which was tapered over two weeks. His symptoms resolved and he is followed up in the outpatient clinic.

## 3. Discussion

There is a dearth of medical literature on the true incidence and etiology of MRS, a disorder with the triad of recurrent facial palsy, furrowed tongue, and orofacial swelling [1, 6, 7]. Majority of patients with this condition will not present for medical evaluation with the classical triad [2, 9, 11]. This condition has been reported in different countries in the world and does not have gender predilection [2, 11]. This condition affects any age group, but reports have shown that patients between 25 and 40 years are most affected [5, 6]. Medical literature suggests that genetics, infections, family history, food allergies, or inflammation may be implicated, but evidence to support this is lacking [3, 8]. Facial nerve weakness like in this patient may occur after months or years of facial swelling; however, our patient had his index episode of orofacial swelling after a two-year history of recurrent lower motor neuron type facial nerve palsy [2–5, 7]. Facial palsy may present on one or both sides of the face. Facial nerve involvement may become permanent following multiple episodes of MRS [2–5, 7]. It has been found in more than 30% of MRS cases [2–5]. In MRS, facial nerve supply is affected and function may worsen after multiple attacks and multiple treatment modalities [2–5, 7].

Diagnosing MRS is very difficult; patients may have had several episodes of one or more of the symptoms before making a definitive diagnosis [2, 6]. Our patient had recurrent episodes of lower motor neuron type facial palsy over a two-year span. MRS may present alone or in conjunction with other chronic diseases like Down's syndrome, sarcoidosis, Crohn's disease, psoriasis, thyroiditis, leprosy, multiple sclerosis, keratitis, ocular palsies, and diabetes mellitus [3]. These relationships are not very clear and are not fully understood; however, some studies have suggested that there might be an autosomal dominant relationship [1, 3].

To address this patient's condition, a thorough history, physical examination, and diagnostic workup were required to make a diagnosis; however, nothing was revealed after extensive workup [8]. The clincher to the diagnosis was a history of recurrent lower motor neuron type facial nerve palsy with right orofacial swelling in this clinic visit; a symptom he had never presented with before. For patients that continue to demonstrate persistence of symptomatology, there is a tendency that resolution may be unlikely; however, many patients have spontaneous resolution of their symptoms [2]. Diagnostic investigations including histology may not be necessary because MRS is a clinical syndrome [11]. CT scan of the head, MRI of the brain, and Chest X-ray may be necessary to rule out associated conditions [3, 4]. In our patient, our top six differential diagnoses included sarcoidosis, Crohn's disease, trigeminal neuralgia, pachymeningitis, angioedema, and stroke. Chest X-ray did not reveal hilar or mediastinal nodal enlargement. A colonoscopy was performed to rule out Crohn's disease and the result was normal. The patient did not complain of facial pain or spasms on the affected side of his face so trigeminal neuralgia was ruled out. We also ruled out pachymeningitis because there was no history of chronic headaches, facial pain, loss of vision, or severe vomiting. CT scan of the head did not show evidence of ischemia or hemorrhage which ruled out stroke or transient ischemic attack, and considering his previous history of predominantly recurrent facial palsy rather than orofacial swelling, it was more likely that this index episode of orofacial swelling was not angioedema. We however could not perform an acquired C1 inhibitor deficiency test to confirm this because of the patient's health insurance coverage.

Considering the right facial paresthesia in this patient's case, we considered palsy of both the trigeminal and facial nerves. In this patient, there was paresthesia in the ophthalmic (V1) and maxillary (V2) divisions of the right side of the face. This may be explained by Li et al. where the authors discuss connections between both nerves such as the trigeminal nerve (infraorbital nerve) and the facial nerve (buccal branch); trigeminal nerve (auriculotemporal nerve) and the upper division of the facial nerve (the zygomatic, buccal, and temporal branches); the supraorbital nerve in connection with the facial nerve (zygomatic and temporal branches); trigeminal nerve (mental nerve) with the facial nerve (marginal mandibular branch) and the facial nerve (via the zygomatic, buccal, and marginal mandibular branch) with the buccinator nerve, a branch of the mandibular division of the trigeminal nerve [12]. While studies have shown that hypoglossal, glossopharyngeal, auditory, and olfactory nerves may be implicated in MRS [3, 10], reports have not shown a combined palsy of both the trigeminal and facial nerves. In previous presentations to the clinic for repeated facial nerve palsy, the patient had been subjected to many investigations including antinuclear antibody (ANA) and antineutrophilic cytoplasmic antibody (ANCA) and they were negative. Steroids, steroid-antibiotic combination (minocycline), steroid-antileprosy combination (clofazimine, dapsone) and NSAIDS have been used to treat MRS [3]. Others include danazol, thalidomide, and sulphasalazine [10]. Facial nerve decompression may become necessary for patients with recurrent MRS with facial paralysis [3–5, 10]. Z-plasty and cheiloplasty may be beneficial for some patients; however, intralesional steroids may be required after surgery. Follow-up with these patients as well as a multidisciplinary approach involving the otolaryngologists, dermatologists, and neurologists may also prove to be useful [3, 10]. Quite recently, studies have shown that benzoate and cinnamon may be helpful in some patients [3].

## 4. Conclusion

MRS patients may have one or more of the following: repeated lower motor neuron type facial nerve palsy, orofacial swelling, and fissuring of the tongue. Recurrent facial nerve paralysis and orofacial swelling in a patient who has received multiple outpatient treatments with steroids in the past are causes for suspicion of MRS. Recognizing the condition with or without its classical presentation is very important.
